# The Third Dimension of Reading the Sugar Code by Lectins: Design of Glycoclusters with Cyclic Scaffolds as Tools with the Aim to Define Correlations between Spatial Presentation and Activity

**DOI:** 10.3390/molecules18044026

**Published:** 2013-04-04

**Authors:** Paul V. Murphy, Sabine André, Hans-Joachim Gabius

**Affiliations:** 1 School of Chemistry, National University of Ireland Galway, University Road, Galway, Ireland; 2 Faculty of Veterinary Medicine, Institute of Physiological Chemistry, Ludwig-Maximilians-University, Veterinärstr, 13, 80539 Munich, Germany; E-Mails: S.Andre@tiph.vetmed.uni-muenchen.de (S.A.); gabius@tiph.vetmed.uni-muenchen.de, gabius@lectins.de (H.-J.G.)

**Keywords:** calixarene, cyclodextrin, galectin, glycome, glycopeptide, glycophane

## Abstract

Coding of biological information is not confined to nucleic acids and proteins. Endowed with the highest level of structural versatility among biomolecules, the glycan chains of cellular glycoconjugates are well-suited to generate molecular messages/signals in a minimum of space. The sequence and shape of oligosaccharides as well as spatial aspects of multivalent presentation are assumed to underlie the natural specificity/selectivity that cellular glycans have for endogenous lectins. In order to eventually unravel structure-activity profiles cyclic scaffolds have been used as platforms to produce glycoclusters and afford valuable tools. Using adhesion/growth-regulatory galectins and the pan-galectin ligand lactose as a model, emerging insights into the potential of cyclodextrins, cyclic peptides, calixarenes and glycophanes for this purpose are presented herein. The systematic testing of lectin panels with spatially defined ligand presentations can be considered as a biomimetic means to help clarify the mechanisms, which lead to the exquisite accuracy at which endogenous lectins select their physiological counterreceptors from the complexity of the cellular glycome.

## 1. The Concept of the Sugar Code

The ubiquitous presence of post-translational modifications, especially phosphorylation, teaches the lesson that it is more than its sequence that determines a protein’s activity profile. A covalent conjugation is able to convey new properties to the protein scaffold [1,2]. As a consequence, the range of protein functionality is likely broadened when processed. Sharing a frequent occurrence with phosphorylation, glycosylation, mostly on cell surface and extracellular proteins (from a monosaccharide to long and even highly branched chains), is known to be an integral part of this system of protein substitutions [3,4,5,6,7,8,9,10]. Clinically, the emerging insights into etiology of aberrations caused by congenital diseases of glycosylation and gain-of-glycosylation mutations are story-telling incidences to illustrate that glycans are not merely inert or readily interchangeable appendices for the protein [11,12,13]. Genetic engineering of animal models, too, underscores the essential nature of glycosylation. Serious defects up to embryonic lethality or neonatal death are caused after ablation of *N*-glycan synthesis (for recent review, please see [14]). In fact, these noted associations are already warranting the monitoring of glycan structure in detail, and this work, applying a combination of technically sophisticated methods, has revealed a level of structural complexity not reached by any other type of protein modification [15,16]. 

The intricacies of the underlying enzymatic machinery, with estimates that at least 1% of the genomic coding capacity is reserved for these enzymes [17], together with enormous versatility of regulation to dynamically shift the glycome profile by remodeling, are the means to let glycosylation become a highly refined process [5,6,18,19]. To give instructive examples, the introduction of certain sugars into glycan chains depends not just on one or a few enzymes. Instead, the fucosylation of mammalian glycans, a characteristic of branch ends and the *N*-glycan core, can be accomplished by thirteen transferases [20,21]. Twenty members belong to the family of sialyltransferases, which are also dedicated to generate elaborate glycan termini [22,23]. Next, the physiological fine-tuning depending on the availability of substrates comes into play to give the glycome its shape; the physiological impact of several types of glycoenzymes, for instance, was crucial to explain the role of glycan reprogramming on the pathway of how a tumor suppressor drives malignant cells into anoikis [24]. Nucleotide sugar transporters, too, deserve to be mentioned, harming their activity being another cause of diseases [25]. As a consequence, the glycophenotype, resulting from the interplay of all these components, can be considered as being as characteristic as a fingerprint for a cell, and carbohydrates can be viewed as a chemical platform to encode biological information.

Toward this end, that is to serve as the third alphabet of life alongside nucleotides and amino acids, sugars have exceptional chemical properties. They actually enable the sugar ‘letters’ to reach an unsurpassed level of coding capacity. Although these features are basic in nature, they deserve to be mentioned in this context to raise awareness for how well sugars are adapted to building code words. Structural variability is possible not just by changing the sequence, as in proteins or nucleic acids. Beyond that, the following parameters can be independently altered when turning units (letters) into oligomers (words): the anomeric status, the linkage position between sugar units, the ring size, branching in oligosaccharides and introduction of site-specific substituents such as acetylation, phosphorylation or sulfation [26,27]. When the synthesis of the glycans is finally completed, their presentation by cell surface glycoproteins (and also by the class of glycolipids [28]) brings these determinants into a strategic position for recognition events. In other words, owing to the generation of bioepitopes with a maximum of information in a minimum of space at readily accessible sites functional implications immediately arise. 

In principle, the presence of the glycans can affect protein properties (acting in *cis*) or the sugar epitope constitutes an entirely new site for recognition by respective receptors (acting in *trans*). Intriguingly, a single sugar unit can act like a switch for the glycan’s three-dimensional structure, hereby building a bridge from the descriptive nature of glycophenotyping to what the respective sugar addition (e.g., core substitutions in *N*-glycans) then triggers [29,30,31,32]. One effect is to cover surface regions of the protein so that the glycan’s shape modulates the potential for contacts to other proteins, in oligomerization and other types of inter-protein contacts. Also, stability and trafficking can depend on sugar signals, a wide field to be explored diligently [5,6,9,12,33]. Having herewith introduced the concept of the sugar code, *i.e.* biological information storage by glycans and transfer into effects via different routes, we can proceed to looking at the mentioned translators of the sugar code, *i.e.*, sugar receptors (lectins).

## 2. Glycans as Bioactive Ligands for Lectins

The specificity of carbohydrate recognition originates from the structural complementarity between the sugar and a protein. Hydrogen-bond networks (without or with involvement of water molecules) and C-H/π-interactions between a patch of suitably positioned C-H bonds (e.g., in d-galactose) and a Trp residue cooperate along the way to give a snug fit [34,35,36]. In special cases, the presence of an anionic sugar part (e.g., in sialylated glycans) accounts for ionic bonds. Sensing the distinct mode of presentation of axial/equatorial hydroxyl groups of common “letters” of the sugar alphabet can also involve Ca^2+^-ion(s) and coordination bonds [37]. These recognition modes combined, not onlyoligosaccharides but monosaccharides such as galactose *vs* mannose can readily be distinguished by lectins, as technically simple assays such as inhibition of lectin-mediated haemagglutination attest. To get a feeling for the extent of the physiological range of interactions via glycan recognition it is instructive to delineate the number of different protein folds with the capacity to bind sugars. A small number would indicate this type of recognition to be more a peculiarity than a frequently encountered mechanism. That would mean that the immense potential of the sugar code outlined above would not really be realized.

As the compilation in Table 1 documents, up to 14 different folds have proven capacity for glycan binding. In each case, examples for respective animal/human lectins are given together with information on glycan ligands. The proteins in the different families cover a wide range of activities, on the level of glycan routing and transport, cell adhesion and growth regulation as well as host defense, to give a few examples (for further information, please see [35,36]). Of note, the binding is remarkably specific to the cellular glycoconjugate, which is the target to ensure the correct flow of information. Despite a large number of theoretically possible contact sites, for example β-galactosides, the lectins are indeed capable to home in on particular glycoproteins/glycolipids or glycosaminoglycan sequences, posing the challenge to identify the underlying molecular reasons. Fittingly, physiologic regulation works on both sides of the recognition system for optimal responsiveness, *i.e.*, the presentation of the lectin and of its glycan counterreceptor(s), seen in distinct contexts of cell adhesion or growth regulation [38,39,40,41]. In addition to making a lectin-reactive epitope available by dynamic remodeling or neosynthesis, topological factors appear to play a major role to guide the selection process. On the side of the glycans, six levels of affinity regulation have been identified, which underlie detectable preferences [36]. Spatial vicinity of ligands, as facilitated within microdomains so that perturbation of their integrity harms lectin reactivity [42], is an efficient means to build preferred contact regions, in terms of affinity and the nature of the counterreceptor. Depending on the cell type a particular glycoprotein (such as the α_5_β_1_-integrin) or a ganglioside (GM1) can be the main binding partner for the same endogenous lectin, the association then setting in motion a post-binding signaling cascade, e.g., toward anergy, anoikis or growth arrest [39,40,43]. Of course, the *in situ* constellations operative in turning structure (at each of the six levels mentioned above) into distinct effects set attractive role models for the synthetic design of glycoclusters.

**Table 1:** Overview of folds with capacity to bind sugars and of lectin classes.

Type of fold	Example for lectin	Example for ligand
β-sandwich (jelly-roll)	(a) galectins	β-galactosides
	(b) calnexin, calreticulin	Glc_1_Man_9_Glc*N*Ac_2_
	(c) ERGIC-53, VIP36, VIPL	Man_x_Glc*N*Ac_2_
	(d) CRD ^a^ of Fbs1 in SCF E3 ubiquitin ligase and peptide-*N*-glycanase	Man_3_Glc*N*Ac_2_; mannopentaose
	(e) pentraxins	glycosaminoglycans, MOβDG, 3-sulfated Gal, Gal*N*Ac and GlcA, Man-6-phosphate
	(f) G-domains of the LNS family (laminin, agrin)	heparin
C-type	asialoglycoprotein receptor, collectins, selectins	Fuc, Gal, Gal*N*Ac, Man, heparin tetrasaccharide
I-type (Ig fold)	N-CAM, TIM-3, siglecs	Man_6_Glc*N*Ac_2_, HNK-1 epitope, α2,3/6-sialylated glycans
P-type	mannose-6-phosphate receptors (MR) and proteins with MR homology domain (erlectin, OS-9)	Man-6-phosphate, Man_5,8_Glc*N*Ac_2_
β-trefoil	(a) fibroblast growth factors	heparan sulfate
	(b) cysteine-rich domain of C-type macrophage mannose receptor	Gal*N*Ac-4-sulfate in Lacdi*N*Ac
	(c) lectin domain in Gal*N*Ac-Ts^b^ involved in mucin-type O-glycosylation	Gal*N*Ac
	(d) hemolytic lectin CEL-III of sea cucumber and lectin EW29 of earthworm	Gal
β-propeller	(a) 4-bladed: tachylectin-3	S-type lipopolysaccharide
	(b) 5-bladed: tachylectin-2	Glc*N*Ac/Gal*N*Ac
	(c) 6-bladed: tachylectin-1	KDO
β-propeller	(a) 4-bladed: tachylectin-3	S-type lipopolysaccharide
	(b) 5-bladed: tachylectin-2	Glc*N*Ac/Gal*N*Ac
	(c) 6-bladed: tachylectin-1	KDO
β-prism I	secretory proteins zg16p/b	not defined
β-prism II	pufferfish (fugu) lectin	Man
β-barrel with jelly-roll topology	tachylectin-4, eel (*Anguilla anguilla*) agglutinin, X-epilectin	Fuc
fibrinogen-like domain	(a) ficolins	Glc*N*Ac**
	(b) intelectins (mammalian, *Xenopus*)	Gal*f*, pentoses
	(c) tachylectin-5	*N*-acetylated sugars
	(d) slug (*Limax flavus*) lectin	sialic acid
link module	CD44, TSG-6, LYVE-1, aggregating proteoglycans	hyaluronic acid
hevein-like domain	tachycytin and spider (*Selenocosmia huwena*) neurotoxin; cobra venom cardiotoxin	Gal*N*Ac; heparin-derived disaccharide
(β/α)_8_ barrel(glycoside hydrolase family 18)	YKL-40 (human cartilage glycoprotein-39; chitinase-like lectin)	(Glc*N*Ac)_n_
short consensus repeat(complement control protein module)	factor H (complement regulator)	glycosaminoglycans, sialic acid

^a ^carbohydrate recognition domain, ^b^*N*-acetylgalactosaminyltransferases; adapted from [44], with permission.

The most telling example concerns the first mammalian lectin purified from rabbit liver, a hepatic receptor acting in clearance of glycoproteins from serum [45]. Testing its glycan reactivity, a geometrical increase of affinity was measured to arise from a numerical increase of valency in oligosaccharides when targeting this C-type lectin (please see also Table 1) [46]. Besides natural or synthetic *N*-glycans, cluster glycosides have been instrumental to trace the intriguing correlation of matching complementarity between ligand and receptor presentation [46,47,48,49,50]. These results led to the definition of the *glycoside cluster effect*, *i.e.*, the affinity enhancement by multivalency over and beyond what is expected from the concentration increase [46,51]. In order to discern rules for the correlation between the topological aspects of ligand presentation and the lectin structure it is reasonable to focus on a certain family of lectins as test model system. For the scope of this review, we do so by dealing exclusively with adhesion/growth-regulatory galectins. Being deliberately placed in the top part of Table 1, these lectins share a β-sandwich fold and a sequence signature with a central Trp residue essential for the C-H/π-interaction with galactose noted above [36], a feature readily monitored using NMR and fluorescence spectroscopy [52,53,54].

## 3. Galectins: a Network of Bioeffectors

**Figure 1:**
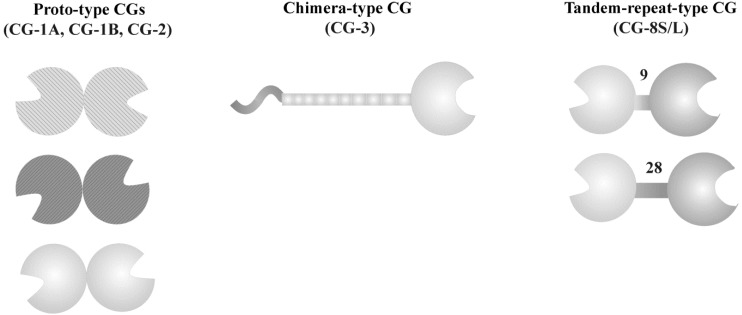
Schematic illustration of the three types of spatial CRD presentation in galectins, using the five chicken galectins as example. The ten Gly/Pro-rich repeats in CG-3 and the lengths of the linker peptide in CG-8 given in number of amino acids are indicated (from [66], with permission).

The common structural traits noted above concern the carbohydrate recognition domain (CRD) of galectins. Since the levels of affinity and specificity for cellular glycans, as well as functionality depend on more than monovalent binding, the active lectin is in general more than a single CRD. Examples of growth regulation by human galectin-1, given above [39,40,41,43], show that this lectin can be expected to initiate signaling by cross-linking of counterreceptors, and, indeed, its structure is homodimeric [55]. Within the family of galectins, the relative spatial arrangement of CRDs divides these proteins into three groups: the homodimeric (proto-type) and the tandem-repeat-type family members, the latter with a linker peptide connecting two different CRDs, along with the chimera-type galectin-3 which harbors an N-terminal peptide with sites for Ser phosphorylation and collagen-like repeats enabling oligmerization in the presence of multivalent ligands [56,57,58] (Figure 1). Thus, the capacity of individual proteins to cross-link counterreceptors, as measured in precipitation analysis with a multivalent glycoprotein [59], and the stoichiometry of the complexes is expected to be different so that even functional competition can be predicted. Apparently, this is the case in blocking galectin-1’s growth-inhibitory activity on neuroblastoma and pancreatic cancer cells by the chimera-type galectin-3 [60,61]. Although different in spatial CRD display, the two lectins share specificity to the same glycoconjugate in these cells. Galectin-3 hereby precludes galectin-1 binding but fails to trigger the post-binding signaling leading to growth arrest in the tested cell systems. Because galectins physiologically form a complex network, with different proteins expressed for example in tumors [58,62,63,64,65], it is a pertinent issue to establish individual structure-activity profiles. Keeping the sugar structure constant by using the pan-galectin ligand lactose (or *N*-acetyllactosamine, Lac*N*Ac), glycoclusters are suited for this project line.

**Figure 2:**
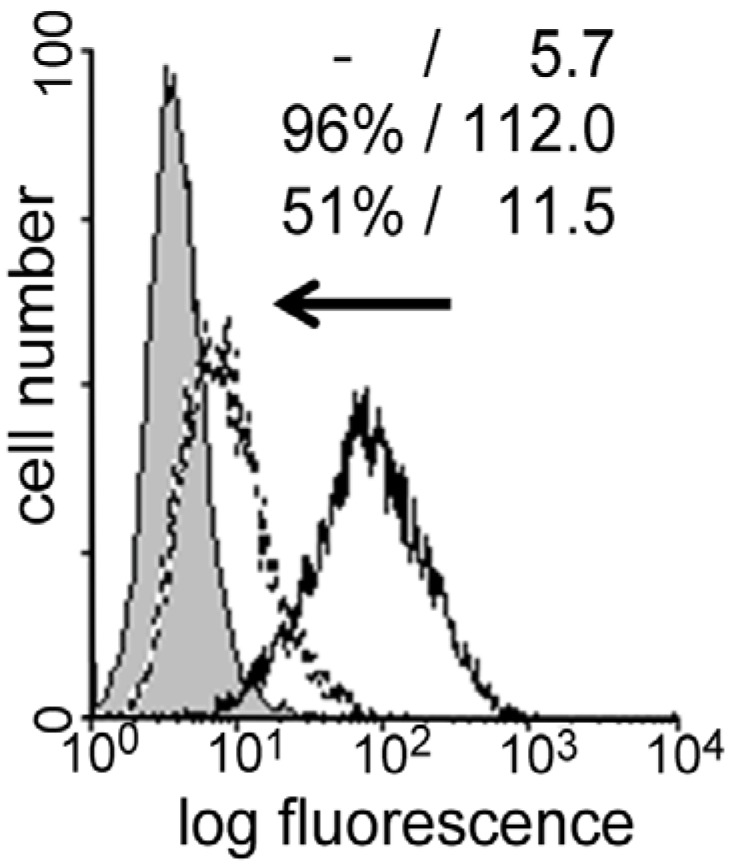
Schematic illustration of the principle of the experimental read-out in cytofluorometric analysis of glycocluster activity. When a labeled lectin binds to cell surfaces, the signal (in percentage of positive cells and mean fluorescence intensity) describes the cells’ reactivity (black line). The presence of an inhibitor reduces staining (dotted line), shifting the binding profile into the direction of the background value (grey area).

**Figure 3:**
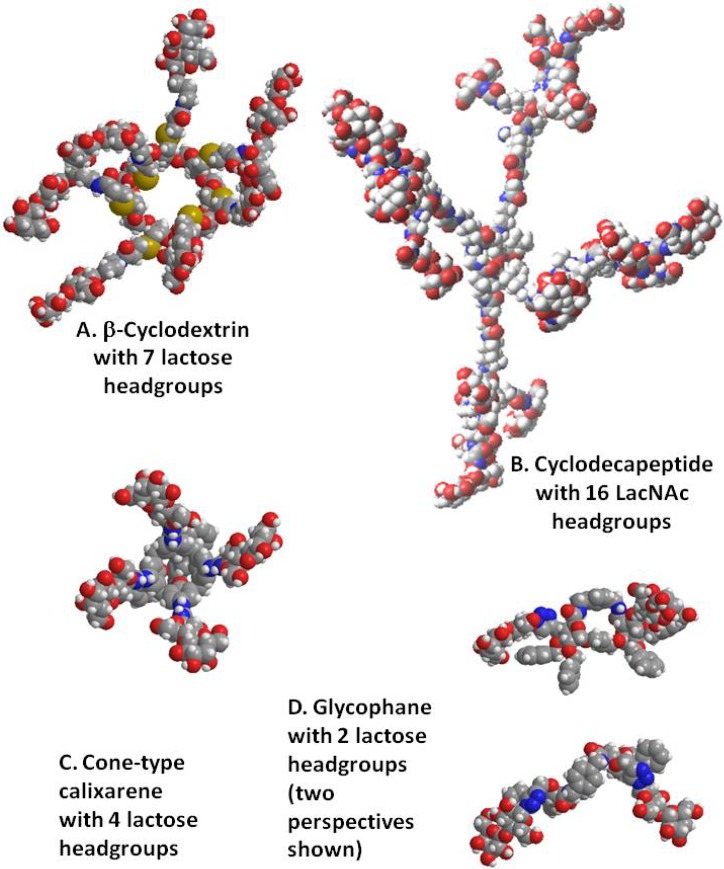
Schematic illustration of the structures of four cyclic scaffolds tested for galectin reactivity, *i.e.*, β-cyclodextrin, cyclic decapeptide, *cone*-type calixarene and glycophane.

Given the different degrees of intra-group diversification in phylogenesis, these experimental series can be run with the complex set of mammalian lectins, to connect the data to biomedical considerations, or, benefiting from organisms having a comparatively low number of galectin genes, with all proteins of such a restricted set of proteins. As shown in Figure 1, five galectins establish the entire panel of chicken galectins (CG), with representatives of each group included, *i.e.*, three homodimeric, one chimera-type and one tandem-repeat-type protein expressed with two linker lengths due to alternative splicing [58,67,68,69,70,71,72]. Regardless of the origin of the galectins the parameter measured in assays with the synthetic compounds is the inhibitory capacity of glycoclusters on the extent of galectin binding to a glycan-presenting matrix. Noting that galectins can be secreted and exert activity as lectins in auto- or paracrine manners on the level of the cell surface (e.g., in tumor growth regulation or in communication between effector and regulatory T cells [73]), the galectin is strictly kept in solution mimicking the physiological situation, while the binding partner is either a glycoprotein (e.g., asialofetuin) adsorbed to the surface of a well of a microtiter plate or a cell surface. The presence of an inhibitor will then reduce the read-out. Cell scanning is performed in cytofluorometric analysis and monitored in terms of percentage of positive cells and mean fluorescence intensity (Figure 2). With galectins as test proteins, four types of cyclic glycoclusters shown in Figure 3 have already been evaluated, with the review of the results herein starting with cyclodextrins.

## 4. Cyclodextrins, Cyclic Decapeptides and Calixarenes

Cyclodextrins are macrocycles of between six to eight α-D-glucose units produced by degradation of starch, which attract attention due to their high biocompatibility and solubility [74,75,76]. Persubstitution of the heptakis 6-deoxy-6-iodo-β-cyclodextrin core was performed with glycosides via their terminal sodium thiolate (for details, please see [77]). The chemical conjugation did not impair the reactivity of the sugar headgroup for lectins. Among the tested galectins, the relatively most sensitive protein was galectin-3 [78], monomeric in solution but capable to pentamerize in the presence of multivalent ligands [79].

The homodimeric galectins-1 and -7 were less responsive [78]. Such a grading had been noted before with a triiodobenzene-based trivalent cluster, to which 2-propynyllactosides had been conjugated [80]. The relative level of inhibition for each protein also depended on the nature of the matrix, here this refers to the type of glycoprotein used and its degree of *N*-glycan branching [78], a result later confirmed when testing other types of glycoclusters [81]. Equally noteworthy, the efficiency of the lactosylated cyclodextrin in interfering with lectin binding was enhanced when tested on galectin-1-presenting cells [78]. Whether the lectin is free in solution or associated to a cell affects the read-out, precluding generalizations. This behaviour with single macrocycles was also observed with a pseudopolyrotaxane-based glycocluster, which had a beads-on-a-string arrangement with lactosylated cyclodextrin being “pearls” on a polyviologen “string” [82,83].

Similarly building on a natural scaffold, cyclic peptides have been decorated with sugar derivatives to generate neoglycopeptide clusters [84,85]. The decapeptides were tailored to have four attachment points for the derivatives via the side chain of the lysine moieties. A clear grading in susceptiblility was seen when moving from galectin-1 to galectins-3 and -4, both in the solid-phase and in the cell assays [86]. Obviously, the presence of the linker in the tandem-repeat-type galectin-4 alters the reactivity to glycoclusters in a bivalent protein, when compared to homodimeric galectin-1. A major affinity difference to the glycoprotein used as matrix had been excluded by calorimetric titrations with human galectins [87]. Since reducing the linker length has consequences for the selection of cell surface ligands [88], the elucidation of the way the linker affects positioning of the two CRDs for cross-linking becomes a topic for further study. Clearly, its presence means more than an increase in inter-CRD distance.

This high level of sensitivity for galectin-4 was also seen with calixarenes [89]. In addition, the *cone-*like tetravalent presentation proved rather discriminatory between galectins-1 and -3. This calixarene display was later confirmed to be mostly inactive for galectin-1 when using an assay based on measuring surface plasmon resonance [90]. The differential reactivity between the two galectins could further be increased by an aromatic 3’-substitution at the galactose unit, yet unfavorably affecting solubility [91]. If it becomes an issue to preclude galectin-3 binding from cells, while maintaining surface binding of galectin-1, such a calixarene (please see Figure 3) with a substituted lactose becomes a possibility, although it is still cross-reactive with tandem-repeat-type members of the galectin family [91]. As a laboratory tool, an assumed functional divergence between galectins-1 and -3 can then be verified without having to resort to manipulations on the genetic level. As seen in Figure 3, a further matrix belongs to the set of macrocycles tested with galectins, *i.e.*, glyco(cyclo)phanes and their acyclic forms. Since this compound class has only recently begun to be explored for lectin reactivity, we add information on reactivity to a plant lectin when mannose is conjugated.

## 5. Glyco(cyclo)phanes

**Figure 4:**
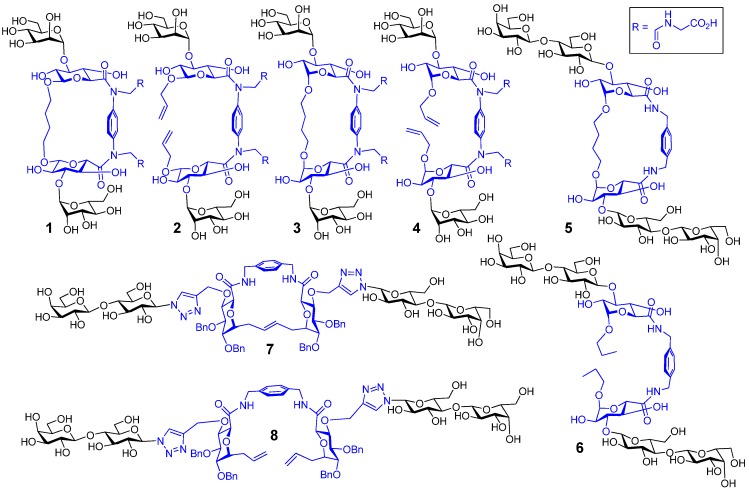
Structures of macrocyclic glycophane scaffolds (blue) with mannose (**1**,**3**) or with lactose (**5**,**7**) and their acyclic analogues (**2**,**4**,**6**,**8**).

**Scheme 1:**
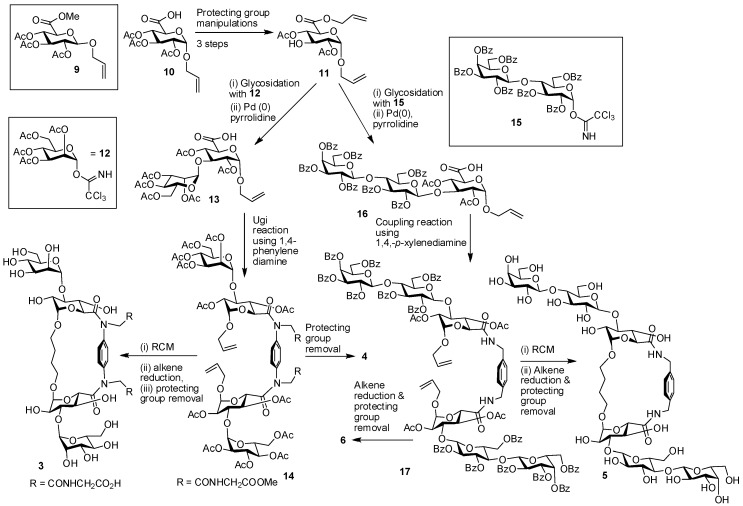
Synthesis of compounds **3**–**6**.

**Scheme 2:**
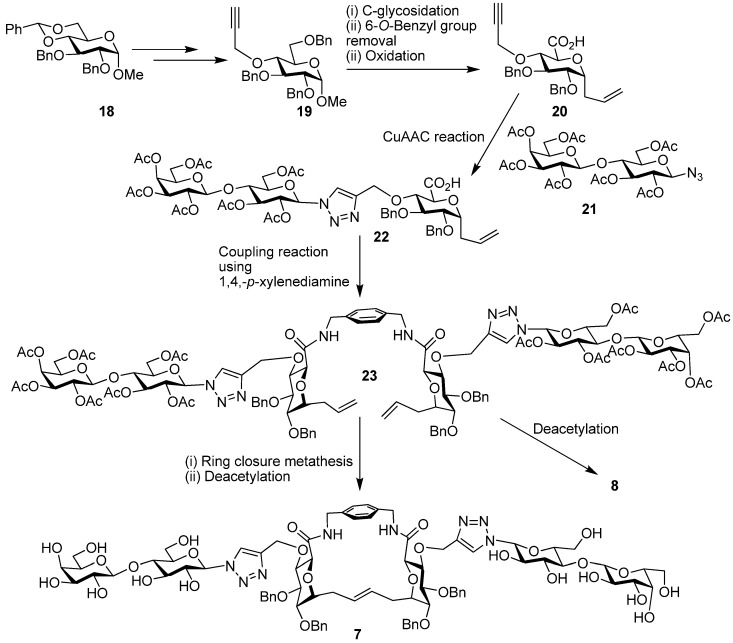
Synthesis of compounds 7 and 8.

Similarly to cyclodextrins, this scaffold has received interest owing to its ability to accommodate guests such as sugars and hereby form inclusion complexes [92,93]. Also found naturally [94], the versatility in the degree of rigidity in the cyclophane scaffold has enabled the proposal of laboratory applications [95,96,97], including the testing of chiral variants of the scaffold for lectin ligand design. Presenting structural details beyond the model in Figure 3, two series of glycophane-based clusters are depicted in Figure 4. They have either a phenylenediamine or a xylylenediamine within the macrocycle, their acyclic analogues also shown, with mannose (**1**–**4**) and lactose (**5**–**8**) grafted to the scaffold as the lectin ligand. The mannose-bearing compounds are included here to illustrate the bioactivity of conjugates beyond galectins. The compounds **1** and **3** are diastereoisomeric glycophanes with α-mannose headgroups, ascertaining the versatility of solid-phase and cell assays as well as testing for differences in relative bioactivities. The saccharides in **1** are linked by a butanediol chain that has β-glycosidic linkages to the glucuronic acid, whereas the linkages to the butanediol chain are α-configured in **3**. Compounds **2** and **4** are the corresponding acyclic analogues of **1** and **3**. The substances **5**–**8** carry β-lactose units for the interaction with galectins, to extend their comparative analysis with cyclic scaffolds. The lactose moieties in **7 **are attached via a triazole-containing linker to position 4 of the glucuronic acid embedded in the glycophane. In **5, **the lactose residues are conjugated directly to the position 3 of the glucuronic acid residue. An overview of the synthetic routes to these products is given in Scheme 1 for compounds **3**–**6** (compounds **1** and **2** were prepared in an analogous manner from β-glucuronide **9**) and Scheme 2 for compounds **7** and **8**. 

The synthesis commenced from the α-glucuronide **10** [98]; its deacetylation followed by formation of an acetylated 6,3-lactone intermediate that was subsequently reacted with allyl alcohol, afforded the glycosyl acceptor **11**. The α-mannose residue was attached to the glucuronic acid derivative by glycosidation with trichloroacetimidate **12**, and subsequent Pd(0)-catalyzed removal of the allyl ester gave **13**. Next, the Ugi reaction [99,100] was employed to produce **14**; this was achieved in good yield (> 80%) in one pot by reacting **13 **with phenylenediamine, formaldehyde and methyl isocyanoacetate. Removal of the acetate-protecting groups from **14** gave acyclic compound **4**. Alternatively, ring closure metathesis (RCM) followed by alkene reduction and acetate removal led to ring closure, thus to glycophane **3**. The diastereoisomers **1** and **2** were prepared from β-glucuronide **9 **by the same route of processing described for **3** and **4**. 

The acceptor **11** proved useful also in the synthesis of **5** and **6** [101]. Glycosidation with benzoylated lactose derivative **15** and subsequent allyl ester hydrolysis made **16 **available. The coupling reaction of **16 **with 1,4-xylylenediamine produced **17**. Reduction and removal of the benzoyl groups generated **6**, while RCM, alkene reduction and benzoyl group removal established the glycophane **5**. As outlined in Scheme 2, the synthesis of compounds **7** and **8** was achieved from alkyne derivative **19**, which was prepared according to literature procedures from the benzylidene **18** [66]. Alkyne **19 **was converted to **20** in three steps, and then the copper-catalyzed azide alkyne cycloaddition (CuAAC) reaction [102] with azide **21** gave **22**. Coupling using 1,4-xylylenediamine led to **23**, which on deacetylation resulted in **8**. When **23** was subjected to RCM [103,104] and the acetates were subsequently removed, this processing established the glycophane derivative **7**. Overall, these glycophane based compounds are bivalent, without/with cyclization and it was postulated that they have different degrees of spatial flexibility for presenting the attached sugars. As a test case for this class of compounds, we here present information on modeling to sample a range of conformations and thus spatial headgroup constellations. 

**Figure 5:**
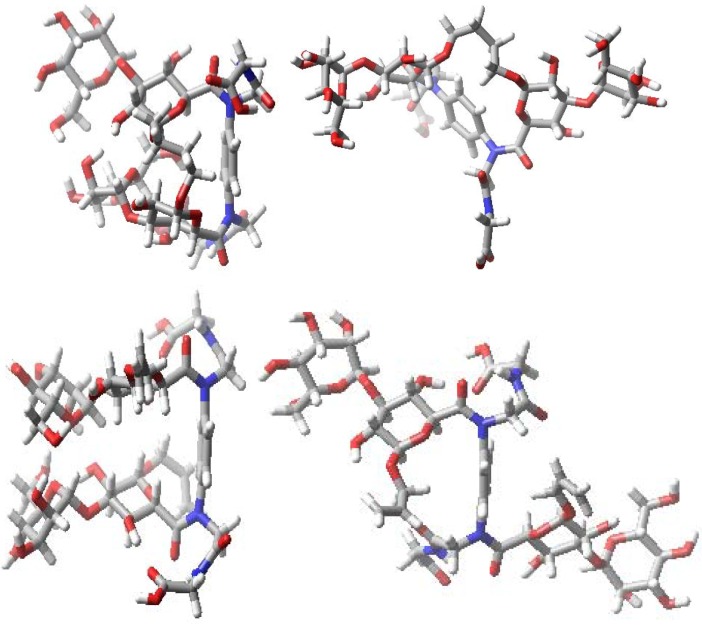
Stacked (left) and extended (right) conformers that can potentially be accessed by themannose-presenting macrocyclic (top) and acyclic (bottom) glycoclusters **1** and **2**.

Conformational searching techniques based on the previously reported approach using Macromodel 8.0 to both compounds **1** and **2** [105,106] suggested that the lowest energy structures are conformers, where the two carbohydrate residues are stacked (Figure 5). It seemed unlikely due to the closeness of the headgroups that lectins would find access to such stacked sugars. Hence, the occurrence of extended conformers of both the glycophanes and their acyclic analogues in the trajectories were investigated by molecular dynamics simulations with Macromodel 8.0 (Schrodinger Inc., LLC, New York, NY, USA), to complement the earlier study [106].

**Figure 6:**
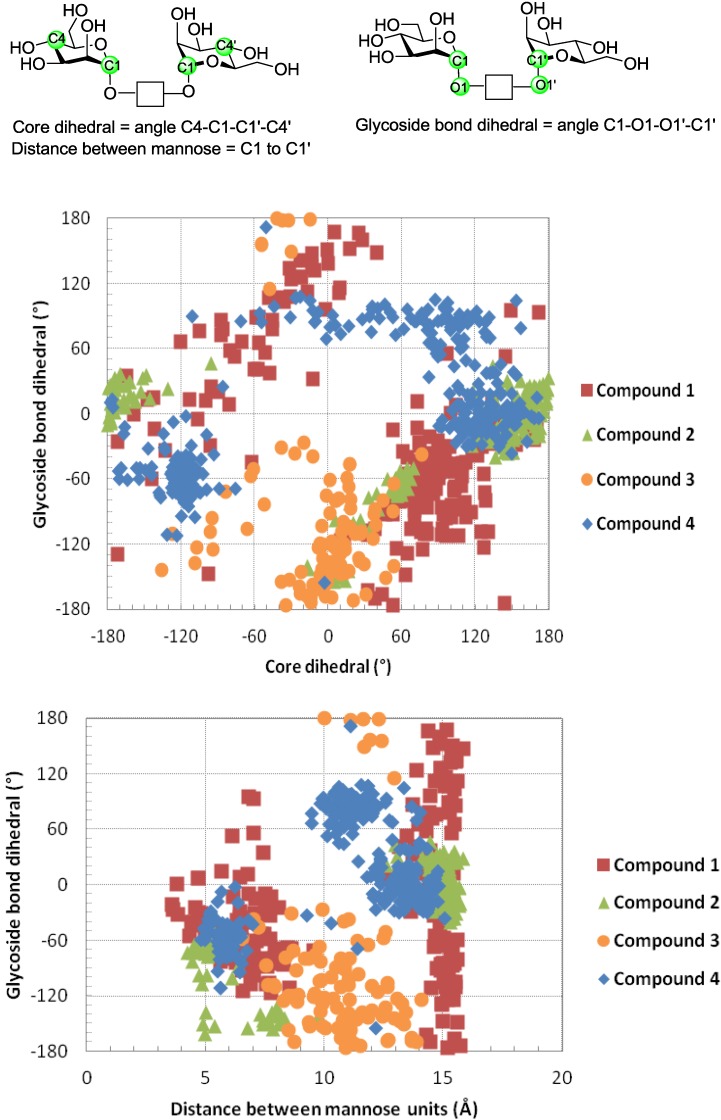
Scatter plots of data for selected conformers of compounds **1**–**4** as generated from molecular dynamics simulations using Macromodel 8.0. The definitions of distance between mannose units, glycoside bond dihedral and core dihedral are shown at the top.

In general, stochastic dynamics was applied to a selected conformer at a temperature of 300 K with an equilibration time of 1 µs and a time step of 1.5 fs using the OPLS-AA force field in the gas phase. Before commencing the simulations for **1**–**4**, the peptide side chains were also extended away from the carbohydrate to exclude forming hydrogen bonding with the carbohydrate residues. During each of the subsequent simulations 100–200 structures were sampled and an internal coordinate system established for determining three spatial parameters (Figure 6, top part). In detail, these were the distances between mannose anomeric carbon atoms (Å), a core dihedral, which is defined by atoms C-4 to C-1 to C-1 to C-4 of the mannose residues, and a glycosidic bond dihedral, which is defined by C1 to O1 to O1′ to C1′ of the mannose residues; the latter two parameters were obtained in order to reach a representation of the relative orientation of the mannose residues. Following these definitions of the coordinate system, the scatter plots which were generated are shown in Figure 6.

These plots illustrate spatial arrangements accessed by the divalent mannosides **1**–**4**. The panels in Figure 6 report that structures, in which the intermannose distance is <8 Å, are often stacked conformers, whereas those with a distance >10 Å can be considered to represent extended conformations. While some overlapping features are observed when comparing the scatter data for the different molecules, each has its own profile.

For example, macrocycle **1** and its acyclic counterpart **2** can access conformations, where the distance between mannose residues is ~15 Å and the glycoside bond dihedral angle varies from −50° to +50°. However, the core dihedral can change from 50°–150° for compound **1 **and from 120° to −160° for compound **2**. Thus, macrocyclization appears to induce a different core orientation between the two mannose residues, even though the distance between these residues and the glycoside bond dihedral may be similar in both the macrocyclic and the acyclic structures. When comparing both macrocycles **1** and **3**, it is clear that the distance between mannose residues differs. The distances were found to vary from 6–14 Å for **3** but more restrained for **1** between 14–16 Å during the simulations; the increased rigidity of **1** compared to **3** can be viewed in respective movies of the dynamics simulations (for access, please go to http://youtu.be/yzZxCGNQ6j0 for compound **1** and http://youtu.be/RZObaf6MD24 for compound **3**).

In terms of bioactivity, the glycoclusters based on mannose were shown to be active as ligands for two leguminous lectins, with cyclization accounting for a trend toward enhanced activity [106]. As testing cells with shifts in the glycome revealed, the specific nature of the glycan display has a marked bearing on relative levels of inhibition [106]. Such an impact of structural aspects of glycosylation had been noted before when examining properties of different glycoproteins with complex-type *N*-glycosylation in the solid-phase assay on galectins [78,81]. These observations preclude extrapolations and require the establishment of a broad experimental basis. They also attest reactivity of the presented mannose moieties to the plant lectins, encouraging work on the lactosides and galectins.

**Figure 7:**
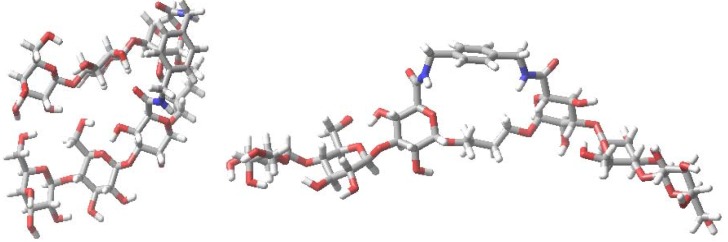
Examples of stacked (left) and extended (right) conformers of the lactose-presenting macrocyclic glycocluster **5**.

**Figure 8:**
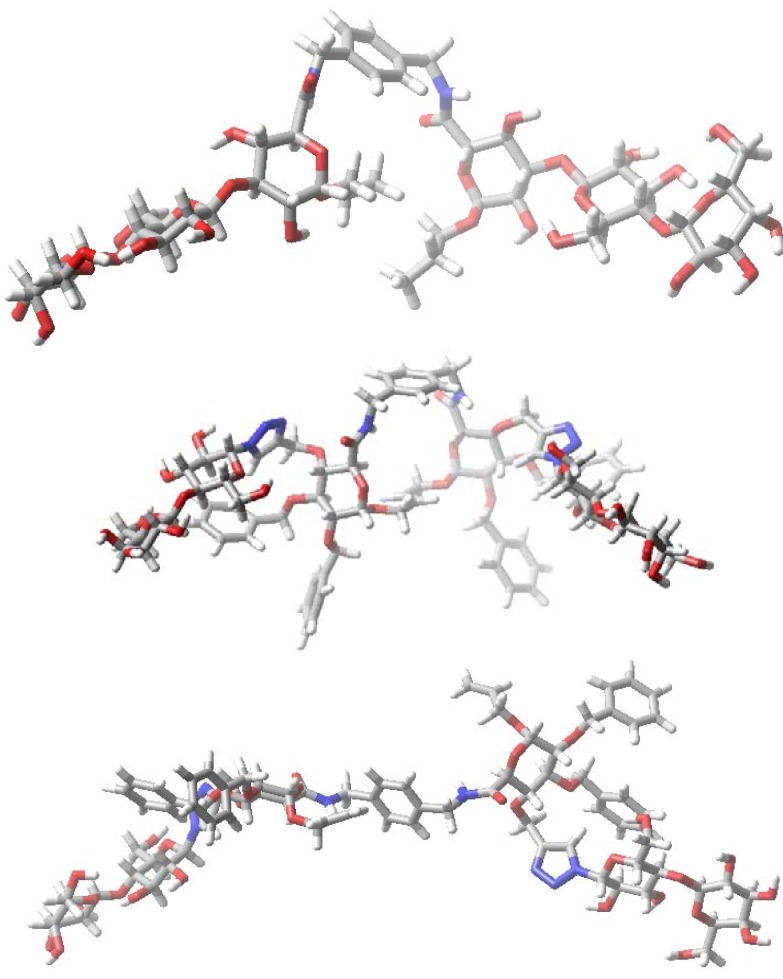
Models of lactose-bearing compounds **6** (top), **7** (middle) and **8** (bottom) in extended conformations.

**Figure 9:**
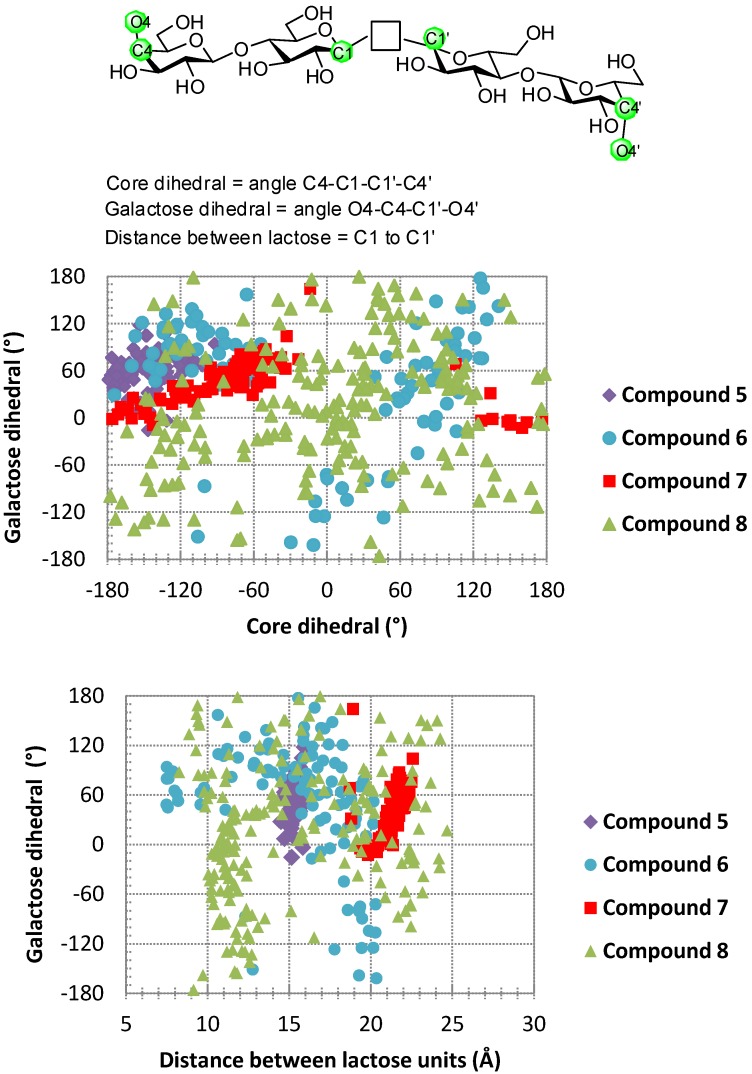
Spatial parameters for extended conformers of compounds **5**–**8**. The definition for the galactose dihedral, core structure and distance between lactose units is shown on top.

Running the same protocols for the lactose-presenting compounds **5**–**8** containing 1,4-xylylenediamine as opposed to the phenylenediamine unit in compounds **1**–**4 **also led to stacked conformers. To interact with galectins extended conformations (Figure 7 and Figure 8) may be more relevant. In this case of lactose, the core dihedral was defined by atoms Gal C-4 to Glc C-1 to Glc C-1′ to Gal C-4′ of the lactose residues, the galactose dihedral is the dihedral angle defined by Gal O-4 to Gal C-4 to Gal C-4′ to Gal O-4′ and the distance is that measured between the two anomeric carbon atoms of glucose (Figure 9). The scatter plots illustrate spatial arrangements accessible to the divalent lactosides **5**–**8**, excluding stacked conformers. At inter-lactose distances of >10 Å extended conformations will be reached. As noted above, each compound has its own conformational profile. Both macrocyclic compounds showed more rigidity in terms of distance between the lactose residues when compared with the acyclic analogues; the distance between lactose residues in compound **5** varied from 14–16 Å, increasing to 19–23 Å for **7**, what reflects the impact of linker characteristics. The extended conformers for the acyclic analogues can apparently reach inter-lactose distances ranging from 10 Å to 25 Å. 

In terms of bioactivity, the glycophane **5** was markedly more active against the CRD of human galectin-3, a product of proteolytic truncation, than the full-length protein [101]. The spatial characteristics of the cyclic form **5** facilitated selectivity between galectin-3 and its truncated version at a discriminatory level of an about 5fold difference [101]. The disparity between the cyclic and acyclic compounds **5** and **6** for this protein is more than 10fold, the IC_50_-values at 0.3 mM and 5 mM, respectively. The cyclic form **7** was found to be more active for CG-8, the chicken tandem-repeat-type galectin, than its acyclic analogue **8**, the increase of relative inhibitory capacity to lactose being 4.5 fold [66]. The triazole at the anomeric carbon appears to be a favorable linker part, as corroborated with other types of glycoclusters [107]. Overall, these data and the examples from the previous paragraphs document the activity of cyclic glycoclusters, giving reason to add comments on ways to enhance their inhibitory capacity/selectivity and the potential for applications. 

## 6. How to Optimize Inhibition, What to Expect

Two main parameter changes are possible in order to increase the inhibitory capacity of a glycocluster on the lectin-glycan interaction, beyond the spatial features discussed so far in this review. The first structural region for tailoring is the aglyconic anomeric extension. It can strengthen binding of the sugar headgroup and provides the functional group for attachment to the scaffold. Experience with galectins, starting with detecting the slight enhancing effect of reactivity of p-aminophenyllactoside relative to lactose for galectin-1 [108,109] and further exploring other aglyconic substituents, revealed quantitative effects not above a several-fold increase [110,111]. More strongly, the nature of the sugar headgroup can exert an effect on affinity and selectivity, the second parameter change. Respective systematic profiling, for example by binding and inhibition assays using glycoproteins/glycan derivatives and free or resin-immobilized galectins, even as isotopically labeled probes in NMR spectroscopy, by docking protocols, titration calorimetry or glycan arrays documented the significance of this factor [112,113,114,115,116,117,118,119,120,121]. Exploiting this parameter holds the promise to accomplish improvements. Already a rather minor biomimetic adaptation such as taking advantage of the physiologic target specificity of galectin-4 for the sulfatide headgroup in apical delivery of glycoproteins [122,123] can likely be very helpful.This teamed up with dendrimeric display, taking into account this lectin’s sensitivity for high ligand density [86,89,124], offers a tempting perspective. Combining the power and creativity of synthetic carbohydrate chemistry [125] and the well-studied chemical routes toward multivalent display [126], for example in starburst dendrimers tailored to galectin-1 [127], with comprehensive case-by-case testing of galectin panels is expected to track down any discriminatory ability that glycoclusters have for these (and other) lectins. At the same time, the affinity increase by cluster design can be conducive to let it act as postal address for cargo delivery by bitopic conjugates, e.g., directing a phototoxin (hematoporphyrin) or inhibitors of matrix metalloproteinases to sites of high galectin density [128,129]. In addition to the cytofluorometric assays cyto- and histochemical determination of staining by labeled galectins [130,131] in the absence/presence of inhibitors will also define their inhibitory potential on lectin binding to physiological counterreceptors. Of note, here changes in signal intensity can be monitored at the membrane or the extracellular space, and also in the cytoplasm and nucleus, providing a sensor for the influence of glycan on intracellular lectin interactions.

Finding optimal combinations of scaffold, linker and sugar headgroup together with its display and favorable dynamics will be helpful to relate spatial aspects of functionality. Considering a perspective for lectin blocking, such parameters will be necessary to minimize cross-reactivity among lectins of a family and also between different lectin families sharing carbohydrate specificity, a major caveat not to be neglected before claiming any application of lectin-targeted drug design [132]. Moreover, galectins are known as multifunctional proteins, the exact nature of the effect depending on the context. Taking galectin-1 as example, it can be growth inhibitory on carcinoma cells [39,43] but favor tumor progression and invasion not only in glioblastoma but also pancreatic carcinoma [133,134], at the same time for example serving as versatile immune regulator [73]. As discussed in detail previously, caution needs to be exercised and very detailed insights into the multifaceted galectin functionality at the different sites of localization gained before deliberating to interfere with binding of a certain galectin, within the natural network and also in the context of other lectins [132].

## 7. Conclusions

The growing insights into the way lectin-glycan recognition contributes to cell physiology have inspired chemists to design biomimetic glycoclusters. Tested primarily with plant lectins such as concanavalin A or peanut agglutinin as models, covalent conjugation of sugar derivatives to diverse scaffolds has been shown to retain the bioactivity of the sugar headgroup. The enhancement of avidity by cluster formation in neoglycoconjugates has been exploited for different applications, among them directing cargo to cells or lectin localization in tissues and cells [51,135,136,137]. Generally, different modes of CRD presentation are found among lectins, within a protein a tandem-repeat display or even widely separated domains and in non-covalent aggregates, as illustrated in Figure 1 for galectins or for C-type lectins in [138]. This spatial parameter, together with a matching display on the glycan side, is assumed to guide complex formation. The cross-linking is the prerequisite to start signaling for growth control, two examples for the biochemical details of the intracellular cascade presented in [139,140]. Evidently, by making the design of a range of glycan displays possible, glycoclusters become highly welcome tools for delineating structure-activity profiles. In molecular detail, spatial aspects can then be examined in assays of increasing biorelevance. 

Presented data for lactose-bearing glycoclusters revealing respective differences between galectin-1 *vs.* galectins-3 and -4 substantiated that the mode of spatial presentation can markedly matter. Whether this line of research can be viewed to have a therapeutic perspective critically depends on collecting a wealth of information not just on one or few proteins but on the complexity of (a) the natural lectin network, (b) the inherent multifunctionality of its individual members and (c) the glycome, on the mentioned six levels of affinity regulation. Undoubtedly, the synthetic compounds will have their merit in laboratory experiments to relate spatial presentation to reactivity, a key source of specificity/selectivity in translating the sugar code.

## References

[1] Bond A.E., Row P.E., Dudley E. (2011). Post-translation modification of proteins; Methodologies and applications in plant sciences. Phytochemistry.

[2] Hunter T. (2012). Why nature chose phosphate to modify proteins. Phil. Trans. R. Soc. B.

[3] Reuter G., Gabius H.-J. (1999). Eukaryotic glycosylation: whim of nature or multipurpose tool?. Cell. Mol. Life Sci..

[4] Spiro R.G. (2002). Protein glycosylation: Nature, Distribution, Enzymatic formation, And disease implications of glycopeptide bonds. Glycobiology.

[5] Zuber C., Roth J., Gabius H.-J. (2009). *N*-Glycosylation. The Sugar Code. Fundamentals of Glycosciences.

[6] Patsos G., Corfield A., Gabius H.-J. (2009). *O*-Glycosylation: Structural diversity and function. The Sugar Code. Fundamentals of Glycosciences.

[7] Wilson I.B.H., Paschinger H., Rendic D., Gabius H.-J. (2009). Glycosylation of model and “lower” organisms. The Sugar Code. Fundamentals of Glycosciences.

[8] Buddecke E., Gabius H.-J. (2009). Proteoglycans. The Sugar Code. Fundamentals of Glycosciences.

[9] Moremen K.W., Tiemeyer M., Nairn A.V. (2012). Vertebrate protein glycosylation: Diversity, Synthesis and function. Nat. Rev. Mol. Cell Biol..

[10] Muthana S.M., Campbell C.T., Gildersleeve J.C. (2012). Modifications of glycans: Biological significance and therapeutic opportunities. ACS Chem. Biol..

[11] Vogt G., Chapgier A., Yang K., Chuzhanova N., Feinberg J., Fieschi C., Boisson-Dupuis S., Alcais A., Filipe-Santos O., Bustamante J. (2005). Gains of glycosylation comprise an unexpectedly large group of pathogenic mutations. Nat. Genet..

[12] Vogt G., Vogt B., Chuzhanova N., Julenius K., Cooper D.N., Casanova J.L. (2007). Gain-of-glycosylation mutations. Curr. Opin. Genet. Dev..

[13] Hennet T., Gabius H.-J. (2009). Diseases of glycosylation. The Sugar Code. Fundamentals of Glycosciences.

[14] Honke K., Taniguchi N., Gabius H.-J. (2009). Animal models to delineate glycan functionality. The Sugar Code. Fundamentals of Glycosciences.

[15] Nakagawa H., Gabius H.-J. (2009). Analytical aspects: Analysis of protein-bound glycans. The Sugar Code. Fundamentals of Glycosciences.

[16] Higgins E. (2010). Carbohydrate analysis throughout the development of a protein therapeutic. Glycoconj. J..

[17] Hansen S.F., Bettler E., Rinnan A., Engelsen S.B., Breton C. (2010). Exploring genomes for glycosyltransferases. Mol. BioSyst..

[18] Gabius H.-J., André S., Kaltner H., Siebert H.-C. (2002). The sugar code: Functional lectinomics. Biochim. Biophys. Acta.

[19] Cummings R.D. (2009). The repertoire of glycan determinants in the human glycome. Mol. BioSyst..

[20] Ma B., Simala-Grant J.L., Taylor D.E. (2006). Fucosylation in prokaryotes and eukaryotes. Glycobiology.

[21] Aplin J.D., Jones C.J. (2012). Fucose, Placental evolution and the glycocode. Glycobiology.

[22] Harduin-Lepers A., Mollicone R., Delannoy P., Oriol R. (2005). The animal sialyltransferases and sialyltransferase-related genes: A phylogenetic approach. Glycobiology.

[23] Takashima S. (2008). Characterization of mouse sialyltransferase genes: Their evolution and diversity. Biosci. Biotechnol. Biochem..

[24] Amano M., Eriksson H., Manning J.C., Detjen K.M., André S., Nishimura S.-I., Lehtiö J., Gabius H.-J. (2012). Tumour suppressor p16(^INK4a^): Anoikis-favouring decrease in *N/O*-glycan/cell surface sialylation by down-regulation of enzymes in sialic acid biosynthesis in tandem in a pancreatic carcinoma model. FEBS J..

[25] Liu L., Hirschberg C.B. (2013). Developmental diseases caused by impaired nucleotide sugar transporters. Glycoconj. J..

[26] Laine R.A., Gabius H.-J., Gabius S. (1997). The information-storing potential of the sugar code. Glycosciences: Status and Perspectives.

[27] Gabius H.-J. (2000). Biological information transfer beyond the genetic code: The sugar code. Naturwissenschaften.

[28] Kopitz J., Gabius H.-J. (2009). Glycolipids. The Sugar Code. Fundamentals of Glycosciences.

[29] Unverzagt C., André S., Seifert J., Kojima S., Fink C., Srikrishna G., Freeze H., Kayser K., Gabius H.-J. (2002). Structure-activity profiles of complex biantennary glycans with core fucosylation and with/without additional α2,3/α2,6-sialylation: Synthesis of neoglycoproteins and their properties in lectin assays, cell binding, and organ uptake. J. Med. Chem..

[30] André S., Unverzagt C., Kojima S., Frank M., Seifert J., Fink C., Kayser K., von der Lieth C.-W., Gabius H.-J. (2004). Determination of modulation of ligand properties of synthetic complex-type biantennary *N*-glycans by introduction of bisecting Glc*N*Ac *in silico*, *in vitro* and *in vivo*. Eur. J. Biochem..

[31] André S., Kozár T., Schuberth R., Unverzagt C., Kojima S., Gabius H.-J. (2007). Substitutions in the *N*-glycan core as regulators of biorecognition: The case of core-fucose and bisecting Glc*N*Ac moieties. Biochemistry.

[32] André S., Kozár T., Kojima S., Unverzagt C., Gabius H.-J. (2009). From structural to functional glycomics: Core substitutions as molecular switches for shape and lectin affinity of *N*-glycans. Biol. Chem..

[33] Gabius H.-J., van de Wouwer M., André S., Villalobo A. (2012). Down-regulation of the epidermal growth factor receptor by altering *N*-glycosylation: Emerging role of β1,4-galactosyltransferases. Anticancer Res..

[34] Quiocho F.A. (1986). Carbohydrate-binding proteins: Tertiary structures and protein-sugar interactions. Annu. Rev. Biochem..

[35] Lis H., Sharon N. (1998). Lectins: Carbohydrate-specific proteins that mediate cellular recognition. Chem. Rev..

[36] Gabius H.-J., André S., Jiménez-Barbero J., Romero A., Solís D. (2011). From lectin structure to functional glycomics: Principles of the sugar code. Trends Biochem. Sci..

[37] Gabius H.-J. (2011). The how and why of Ca^2+^ involvement in lectin activity. Trends Glycosci. Glycotechnol..

[38] Sperandio M. (2006). Selectins and glycosyltransferases in leukocyte rolling *in vivo*. FEBS J..

[39] André S., Sanchez-Ruderisch H., Nakagawa H., Buchholz M., Kopitz J., Forberich P., Kemmner W., Böck C., Deguchi K., Detjen K.M. (2007). Tumor suppressor p16^INK4a^: Modulator of glycomic profile and galectin-1 expression to increase susceptibility to carbohydrate-dependent induction of anoikis in pancreatic carcinoma cells. FEBS J..

[40] Wang J., Lu Z.H., Gabius H.-J., Rohowsky-Kochan C., Ledeen R.W., Wu G. (2009). Cross-linking of GM1 ganglioside by galectin-1 mediates regulatory T cell activity involving TRPC5 channel activation: Possible role in suppressing experimental autoimmune encephalomyelitis. J. Immunol..

[41] Wu G., Lu Z.H., Gabius H.-J., Ledeen R.W., Bleich D. (2011). Ganglioside GM1 deficiency in effector T cells from NOD mice induces resistance to regulatory T cell suppression. Diabetes.

[42] Kopitz J., Bergmann M., Gabius H.-J. (2010). How adhesion/growth-regulatory galectins-1 and -3 attain cell specificity: Case study defining their target on neuroblastoma cells (SK-N-MC) and marked affinity regulation by affecting microdomain organization of the membrane. IUBMB Life.

[43] Fischer C., Sanchez-Ruderisch H., Welzel M., Wiedenmann B., Sakai T., André S., Gabius H.-J., Khachigian L., Detjen K., Rosewicz S. (2005). Galectin-1 interacts with the α_5_β_1_ fibronectin receptor to restrict carcinoma cell growth via induction of p21 and p27. J. Biol. Chem..

[44] Gabius H.-J., Gabius H.-J. (2009). Animal and human lectins. The Sugar Code. Fundamentals of Glycosciences.

[45] Hudgin R.L., Pricer W.E., Ashwell G., Stockert R.J., Morell A.G. (1974). The isolation and properties of a rabbit liver binding protein specific for asialoglycoproteins. J. Biol. Chem..

[46] Lee Y.C., Townsend R.R., Hardy M.R., Lönngren J., Arnarp J., Haraldsson M., Lönn H. (1983). Binding of synthetic oligosaccharides to the hepatic Gal/Gal*N*Ac lectin. J. Biol. Chem..

[47] Rogers J.C., Kornfeld S. (1971). Hepatic uptake of proteins coupled to fetuin glycopeptide. Biochem. Biophys. Res. Commun..

[48] Lee R.T., Lin P., Lee Y.C. (1984). New synthetic cluster ligands for galactose/*N*-acetylgalactosamine-specific lectin of mammalian liver. Biochemistry.

[49] Lee R.T., Lee Y.C. (1987). Preparation of cluster glycosides of *N*-acetylgalactosamine that have subnanomolar binding constants towards the mammalian hepatic Gal/Gal*N*Ac-specific receptor. Glycoconj. J..

[50] André S., Kojima S., Gundel G., Russwurm R., Schratt X., Unverzagt C., Gabius H.-J. (2006). Branching mode in complex-type triantennary *N*-glycans as regulatory element of their ligand properties. Biochim. Biophys. Acta.

[51] Lee R.T., Lee Y.C., Lee Y.C., LeeR T. (1994). Enhanced biochemical affinities of multivalent neoglycoconjugates. Neoglycoconjugates. Preparation and Applications.

[52] Siebert H.-C., Adar R., Arango R., Burchert M., Kaltner H., Kayser G., Tajkhorshid E., von der Lieth C.-W., Kaptein R., Sharon N. (1997). Involvement of laser photo CIDNP (chemically induced dynamic nuclear polarization)-reactive amino acid side chains in ligand binding by galactoside-specific lectins in solution. Eur. J. Biochem..

[53] Göhler A., Büchner C., André S., Doose S., Kaltner H., Gabius H.-J. (2011). Sensing ligand binding to a clinically relevant lectin by tryptophan fluorescence anisotropy. Analyst.

[54] Göhler A., Büchner C., Doose S., Kaltner H., Gabius H.-J. (2012). Analysis of homodimeric avian and human galectins by two methods based on fluorescence spectroscopy: Different structural alterations upon oxidation and ligand binding. Biochimie.

[55] López-Lucendo M.F., Solís D., André S., Hirabayashi J., Kasai K.-I., Kaltner H., Gabius H.-J., Romero A. (2004). Growth-regulatory human galectin-1: Crystallographic characterisation of the structural changes induced by single-site mutations and their impact on the thermodynamics of ligand binding. J. Mol. Biol..

[56] Kasai K.-I., Hirabayashi J. (1996). Galectins: A family of animal lectins that decipher glycocodes. J. Biochem..

[57] Cooper D.N.W. (2002). Galectinomics: Finding themes in complexity. Biochim. Biophys. Acta.

[58] Kaltner H., Gabius H.-J. (2012). A toolbox of lectins for translating the sugar code: the galectin network in phylogenesis and tumors. Histol. Histopathol..

[59] Gupta D., Kaltner H., Dong X., Gabius H.-J., Brewer C.F. (1996). Comparative cross-linking activities of lactose-specific plant and animal lectins and a natural lactose-binding immunoglobulin G fraction from human serum with asialofetuin. Glycobiology.

[60] Kopitz J., von Reitzenstein C., André S., Kaltner H., Uhl J., Ehemann V., Cantz M., Gabius H.-J. (2001). Negative regulation of neuroblastoma cell growth by carbohydrate-dependent surface binding of galectin-1 and functional divergence from galectin-3. J. Biol. Chem..

[61] Sanchez-Ruderisch H., Fischer C., Detjen K.M., Welzel M., Wimmel A., Manning J.C., André S., Gabius H.-J. (2010). Tumor suppressor p16^INK4a^: Downregulation of galectin-3, an endogenous competitor of the pro-anoikis effector galectin-1, in a pancreatic carcinoma model. FEBS J..

[62] Gabius H.-J., Brehler R., Schauer A., Cramer F. (1986). Localization of endogenous lectins in normal human breast, benign breast lesions and mammary carcinomas. Virch. Arch. [Cell. Pathol.].

[63] Kayser K., Höft D., Hufnagl P., Caselitz J., Zick Y., André S., Kaltner H., Gabius H.-J. (2003). Combined analysis of tumor growth pattern and expression of endogenous lectins as a prognostic tool in primary testicular cancer and its lung metastases. Histol. Histopathol..

[64] Saussez S., de Leval L., Decaestecker C., Sirtaine N., Cludts S., Duray A., Chevalier D., André S., Gabius H.-J., Remmelink M., Leroy X. (2010). Galectin fingerprinting in Warthin’s tumors: Lectin-based approach to trace its origin?. Histol. Histopathol..

[65] Remmelink M., de Leval L., Decaestecker C., Duray A., Crompot E., Sirtaine N., Andre S., Kaltner H., Leroy X., Gabius H.J. (2011). Quantitative immunohistochemical fingerprinting of adhesion/growth-regulatory galectins in salivary gland tumours: Divergent profiles with diagnostic potential. Histopathology.

[66] André S., Jarikote D.V., Yan D., Vincenz L., Wang G.N., Kaltner H., Murphy P.V., Gabius H.-J. (2012). Synthesis of bivalent lactosides and their activity as sensors for differences between lectins in inter- and intrafamily comparisons. Bioorg. Med. Chem. Lett..

[67] Den H., Malinzak D.A. (1977). Isolation and properties of β-d-galactoside-specific lectin from chick embryo thigh muscle. J. Biol. Chem..

[68] Beyer E.C., Zweig S.E., Barondes S.H. (1980). Two lactose-binding lectins from chicken tissues. Purified lectin from intestine is different from those in liver and muscle. J. Biol. Chem..

[69] Oda Y., Kasai K.-I. (1983). Purification and characterization of β-galactoside-binding lectin from chick embryonic skin. Biochim. Biophys. Acta.

[70] Kaltner H., Solís D., Kopitz J., Lensch M., Lohr M., Manning J.C., Mürnseer M., Schnölzer M., André S., Sáiz J.L. (2008). Proto-type chicken galectins revisited: Characterization of a third protein with distinctive hydrodynamic behaviour and expression pattern in organs of adult animals. Biochem. J..

[71] Kaltner H., Solís D., André S., Lensch M., Manning J.C., Mürnseer M., Saíz J.L., Gabius H.-J. (2009). Unique chicken tandem-repeat-type galectin: Implications of alternative splicing and a distinct expression profile compared to those of the three proto-type proteins. Biochemistry.

[72] Kaltner H., Kübler D., López-Merino L., Lohr M., Manning J.C., Lensch M., Seidler J., Lehmann W.D., André S., Solís D. (2011). Toward comprehensive analysis of the galectin network in chicken: Unique diversity of galectin-3 and comparison of its localization profile in organs of adult animals to the other four members of this lectin family. Anat. Rec..

[73] Ledeen R.W., Wu G., André S., Bleich D., Huet G., Kaltner H., Kopitz J., Gabius H.-J. (2012). Beyond glycoproteins as galectin counterreceptors: Tumor/effector T cell growth control via ganglioside GM1. Ann. NY Acad. Sci..

[74] Fulton D.A., Stoddart J.F. (2001). Neoglycoconjugates based on cyclodextrins and calixarenes. Bioconjug. Chem..

[75] Houseman B.T., Mrksich M. (2002). Model systems for studying polyvalent carbohydrate binding interactions. Top. Curr. Chem..

[76] Mellet C.O., Defaye J., Fernandez J.M.G. (2002). Multivalent cyclooligosaccharides: Versatile carbohydrate clusters with dual role as molecular receptors and lectin ligands. Chem. Eur. J..

[77] Furuike T., Aiba S., Nishimura S.I. (2000). A highly practical synthesis of cyclodextrin-based glycoclusters having enhanced affinity with lectins. Tetrahedron.

[78] André S., Kaltner H., Furuike T., Nishimura S.-I., Gabius H.-J. (2004). Persubstituted cyclodextrin-based glycoclusters as inhibitors of protein-carbohydrate recognition using purified plant and mammalian lectins and wild-type and lectin-gene-transfected tumor cells as targets. Bioconjug. Chem..

[79] Ahmad N., Gabius H.-J., André S., Kaltner H., Sabesan S., Roy R., Liu B., Macaluso F., Brewer C.F. (2004). Galectin-3 precipitates as a pentamer with synthetic multivalent carbohydrates and forms heterogeneous cross-linked complexes. J. Biol. Chem..

[80] André S., Liu B., Gabius H.-J., Roy R. (2003). First demonstration of differential inhibition of lectin binding by synthetic tri-and tetravalent glycoclusters from cross-coupling of rigidified 2-propynyl lactoside. Org. Biomol. Chem..

[81] André S., Specker D., Bovin N.V., Lensch M., Kaltner H., Gabius H.-J., Wittmann V. (2009). Carbamate-linked lactose: Design of clusters and evidence for selectivity to block binding of human lectins to (neo)glycoproteins with increasing degree of branching and to tumor cells. Bioconjug.e Chem..

[82] Nelson A., Belitsky J.M., Vidal S., Joiner C.S., Baum L.G., Stoddart J.F. (2004). A self-assembled multivalent pseudopolyrotaxane for binding galectin-1. J. Am. Chem. Soc..

[83] Belitsky J.M., Nelson A., Hernandez J.D., Baum L.G., Stoddart J.F. (2007). Multivalent interactions between lectins and supramolecular complexes: Galectin-1 and self-assembled pseudopolyrotaxanes. Chem. Biol..

[84] Wittmann V., Seeberger S. (2000). Combinatorial solid-phase synthesis of multivalent cyclic neoglycopeptides. Angew. Chem. Int. Ed..

[85] Renaudet O. (2008). Recent advances on cyclopeptide-based glycoclusters. Mini-Rev. Org. Chem..

[86] André S., Renaudet O., Bossu I., Dumy P., Gabius H.-J. (2011). Cyclic neoglycodecapeptides: how to increase their inhibitory activity and selectivity on lectin/toxin binding to a glycoprotein and cells. J. Pept. Sci..

[87] Dam T.K., Gabius H.-J., André S., Kaltner H., Lensch M., Brewer C.F. (2005). Galectins bind to the multivalent glycoprotein asialofetuin with enhanced affinities and a gradient of decreasing binding constants. Biochemistry.

[88] Kopitz J., Ballikaya S., André S., Gabius H.-J. (2012). Ganglioside GM1/galectin-dependent growth regulation in human neuroblastoma cells: Special properties of bivalent galectin-4 and significance of linker length for ligand selection. Neurochem. Res..

[89] André S., Sansone F., Kaltner H., Casnati A., Kopitz J., Gabius H.-J., Ungaro R. (2008). Calix[n]arene-based glycoclusters: Bioactivity of thiourea-linked galactose/lactose moieties as inhibitors of binding of medically relevant lectins to a glycoprotein and cell-surface glycoconjugates and selectivity among human adhesion/growth-regulatory galectins. ChemBioChem.

[90] Cecioni S., Matthews S.E., Blanchard H., Praly J.P., Imberty A., Vidal S. (2012). Synthesis of lactosylated glycoclusters and inhibition studies with plant and human lectins. Carbohydr. Res..

[91] André S., Grandjean C., Gautier F.M., Bernardi S., Sansone F., Gabius H.-J., Ungaro R. (2011). Combining carbohydrate substitutions at bioinspired positions with multivalent presentation towards optimising lectin inhibitors: Case study with calixarenes. Chem. Commun. (Camb).

[92] Bukownik R.R., Wilcox C.S. (1988). Synthetic receptors. 3,6-Anhydro-7-benzenesulfonamido-1,7-dideoxy-4,5-O-isopropylidene-D-altro-hept-1-ynitol: A useful component for the preparation of chiral water-soluble cyclophanes based on carbohydrate precursors. J. Org. Chem..

[93] Jiménez-Barbero J., Junquera E., Martin-Pastor M., Sharma S., Vicent C., Penades S.  (1995). Molecular recognition of carbohydrates using a synthetic receptor. A model system to understand the stereoselectivity of a carbohydrate-carbohydrate interaction in water. J. Am. Chem. Soc..

[94] Gulder T., Baran P.S. (2012). Strained cyclophane natural products: Macrocyclization at its limits. Nat. Prod. Rep..

[95] Velasco-Torrijos T., Murphy P.V. (2005). Synthesis and conformational analysis of novel water soluble macrocycles incorporating carbohydrates, including a β-cyclodextrin mimic. Tetrahedron- Asymmetr..

[96] Murphy P.V., Müller-Bunz H., Velasco-Torrijos T. (2005). The crystal structure of a cyclic glycolipid reveals a carbohydrate-carbohydrate interaction interface. Carbohydr. Res..

[97] Murphy P.V. (2007). Peptidomimetics, glycomimetics and scaffolds from carbohydrate building blocks. Eur. J. Org. Chem..

[98] Polakova M., Pitt N., Tosin M., Murphy P.V. (2004). Glycosidation reactions of silyl ethers with conformationally inverted donors derived from glucuronic acid: Stereoselective synthesis of glycosides and 2-deoxyglycosides. Angew. Chem. Int. Ed..

[99] Bradley H., Fitzpatrick G., Glass W.K., Kunz H., Murphy P.V. (2001). Application of Ugi reactions in synthesis of divalent neoglycoconjugates: Evidence that the sugars are presented in restricted conformation. Org. Lett..

[100] Ugi I., Werner B., Dömling A. (2003). The chemistry of isocyanides, their multicomponent reactions and their librarie. Molecules.

[101] Leyden R., Velasco-Torrijos T., André S., Gouin S., Gabius H.-J., Murphy P.V. (2009). Synthesis of bivalent lactosides based on terephthalamide, *N,N'*-diglucosylterephthalamide, and glycophane scaffolds and assessment of their inhibitory capacity on medically relevant lectins. J. Org. Chem..

[102] Tornoe C.W., Christensen C., Meldal M. (2002). Peptidotriazoles on solid phase: [1,2,3]-Triazoles by regiospecific copper(i)-catalyzed 1,3-dipolar cycloadditions of terminal alkynes to azides. J. Org. Chem..

[103] Trnka T.M., Grubbs R.H. (2001). The development of L2X2Ru=CHR olefin metathesis catalysts: An organometallic success story. Acc. Chem. Res..

[104] Jarikote D.V., Murphy P.V. (2010). Metathesis and macrocycles with embedded carbohydrates. Eur. J. Org. Chem..

[105] Mohamadi F., Richards N.G.J., Guida W.C., Liskamp R., Lipton M., Caufield C., Chang G., Hendrickson T., Still W.C. (1990). Macromodel: An integrated software system for modeling organic and bioorganic molecules using molecular mechanics. J. Comput. Chem..

[106] André S., Velasco-Torrijos T., Leyden R., Gouin S., Tosin M., Murphy P.V., Gabius H.-J. (2009). Phenylenediamine-based bivalent glycocyclophanes: Synthesis and analysis of the influence of scaffold rigidity and ligand spacing on lectin binding in cell systems with different glycomic profiles. Org. Biomol. Chem..

[107] Wang G.-N., André S., Gabius H.-J., Murphy P.V. (2012). Bi- to tetravalent glycoclusters: Synthesis, structure-activity profiles as lectin inhibitors and impact of combining both valency and headgroup tailoring on selectivity. Org. Biomol. Chem..

[108] Ahmed H., Allen H.J., Sharma A., Matta K.L. (1990). Human splenic galaptin: Carbohydrate-binding specificity and characterization of the combining site. Biochemistry.

[109] Lee R.T., Ichikawa Y., Allen H.J., Lee Y.C. (1990). Binding characteristics of galactoside-binding lectin (galaptin) from human spleen. J. Biol. Chem..

[110] André S., Giguère D., Dam T.K., Brewer C.F., Gabius H.-J., Roy R. (2010). Synthesis and screening of a small glycomimetic library for inhibitory activity on medically relevant galactoside-specific lectins in assays of increasing biorelevance. New J. Chem..

[111] Giguère D., André S., Bonin M.A., Bellefleur M.A., Provencal A., Cloutier P., Pucci B., Roy R., Gabius H.-J. (2011). Inhibitory potential of chemical substitutions at bioinspired sites of β-*D*-galactopyranose on neoglycoprotein/cell surface binding of two classes of medically relevant lectins. Bioorg. Med. Chem..

[112] Sparrow C., Leffler H., Barondes S.H. (1987). Multiple soluble α-galactoside-binding lectins from human lung. J. Biol. Chem..

[113] Solís D., Romero A., Kaltner H., Gabius H.-J., Díaz-Mauriño T. (1996). Different architecture of the combining sites of two chicken galectins revealed by chemical-mapping studies with synthetic ligand derivatives. J. Biol. Chem..

[114] Ahmad N., Gabius H.-J., Kaltner H., André S., Kuwabara I., Liu F.-T., Oscarson S., Norberg T., Brewer C.F. (2002). Thermodynamic binding studies of cell surface carbohydrate epitopes to galectins-1, -3 and -7. Evidence for differential binding specificities. Can. J. Chem..

[115] Hirabayashi J., Hashidate T., Arata Y., Nishi N., Nakamura T., Hirashima M., Urashima T., Oka T., Futai M., Müller W.E.G. (2002). Oligosaccharide specificity of galectins: A search by frontal affinity chromatography. Biochim. Biophys. Acta.

[116] Stowell S.R., Arthur C.M., Slanina K.A., Horton J.R., Smith D.F., Cummings R.D. (2008). Dimeric Galectin-8 induces phosphatidylserine exposure in leukocytes through polylactosamine recognition by the C-terminal domain. J. Biol. Chem..

[117] Solís D., Maté M.J., Lohr M., Ribeiro J.P., López-Merino L., André S., Buzamet E., Cañada F.J., Kaltner H., Lensch M. (2010). N-Domain of human adhesion/growth-regulatory galectin-9: Preference for distinct conformers and non-sialylated *N*-glycans and detection of ligand-induced structural changes in crystal and solution. Int. J. Biochem. Cell Biol..

[118] Krzeminski M., Singh T., André S., Lensch M., Wu A.M., Bonvin A.M.J.J., Gabius H.-J. (2011). Human galectin-3 (Mac-2 antigen): Defining molecular switches of affinity to natural glycoproteins, structural and dynamic aspects of glycan binding by flexible ligand docking and putative regulatory sequences in the proximal promoter region. Biochim. Biophys. Acta.

[119] Martín-Santamaría S., André S., Buzamet E., Caraballo R., Fernández-Cureses G., Morando M., Ribeiro J.P., Ramírez-Gualito K., de Pascual-Teresa B., Cañada F.J. (2011). Strategic combination of binding studies and NMR spectroscopy and detection of selectivity between a plant toxin and human lectins. Org. Biomol. Chem..

[120] Miller M.C., Ribeiro J.P., Roldós V., Martín-Santamaría S., Cañada F.J., Nesmelova I.V., André S., Pang M., Klyosov A.A., Baum L.G. (2011). Structural aspects of binding of α-linked digalactosides to human galectin-1. Glycobiology.

[121] Vokhmyanina O.A., Rapoport E.M., André S., Severov V.V., Ryzhov I., Pazynina G.V., Korchagina E., Gabius H.-J., Bovin N.V. (2012). Comparative study of the glycan specificities of cell-bound human tandem-repeat-type galectins-4, -8 and -9. Glycobiology.

[122] Delacour D., Gouyer V., Zanetta J.-P., Drobecq H., Leteurtre E., Grard G., Moreau-Hannedouche O., Maes E., Pons A., André S. (2005). Galectin-4 and sulfatides in apical membrane trafficking in enterocyte-like cells. J. Cell Biol..

[123] Stechly L., Morelle W., Dessein A.F., André S., Grard G., Trinel D., Dejonghe M.J., Leteurtre E., Drobecq H., Trugnan G. (2009). Galectin-4-regulated delivery of glycoproteins to the brush border membrane of enterocyte-like cells. Traffic.

[124] Wu A.M., Wu J.H., Liu J.-H., Singh T., André S., Kaltner H., Gabius H.-J. (2004). Effects of polyvalency of glycotopes and natural modifications of human blood group ABH/Lewis sugars at the Galβ1-terminated core saccharides on the binding of domain-I of recombinant tandem-repeat-type galectin-4 from rat gastrointestinal tract (G4-N). Biochimie.

[125] Oscarson S., Gabius H.-J. (2009). The chemist’s way to synthesize glycosides. The Sugar Code. Fundamentals of Glycosciences.

[126] Chabre Y.M., Roy R., Gabius H.-J. (2009). The chemist’s way to prepare multivalency. The Sugar Code. Fundamentals of Glycosciences.

[127] André S., Cejas Ortega P.J., Alamino Perez M., Roy R., Gabius H.-J. (1999). Lactose-containing starburst dendrimers: Influence of dendrimer generation and binding-site orientation of receptors (plant/animal lectins and immunoglobulins) on binding properties. Glycobiology.

[128] Griegel S., Rajewsky M.F., Ciesiolka T., Gabius H.J. (1989). Endogenous sugar receptor (lectin) profiles of human retinoblastoma and retinoblast cell lines analyzed by cytological markers, affinity chromatography and neoglycoprotein-targeted photolysis. Anticancer Res..

[129] Bartoloni M., Dominguez B.E., Dragoni E., Richichi B., Fragai M., André S., Gabius H.-J., Arda A., Luchinat C., Jiménez-Barbero J. (2013). Targeting matrix metalloproteinases: Design of a bifunctional inhibitor for presentation by tumour-associated galectins. Chem. Eur. J..

[130] Habermann F.A., André S., Kaltner H., Kübler D., Sinowatz F., Gabius H.-J. (2011). Galectins as tools for glycan mapping in histology: Comparison of their binding profiles to the bovine zona pellucida by confocal laser scanning microscopy. Histochem. Cell Biol..

[131] Schlötzer-Schrehardt U., André S., Janko C., Kaltner H., Kopitz J., Gabius H.-J., Herrmann M. (2012). Adhesion/growth-regulatory galectins in the human eye: Localization profiles and tissue reactivities as a standard to detect disease-associated alterations. Graefes Arch. Clin. Exp. Ophthalmol..

[132] Smetana K., André S., Kaltner H., Kopitz J., Gabius H.-J. (2013). Context-dependent multifunctionality of galectin-1: A challenge for defining the lectin as therapeutic target. Exp. Opin. Ther. Targ..

[133] Rorive S., Belot N., Decaestecker C., Lefranc F., Gordower L., Micik S., Maurage C.-A., Kaltner H., Ruchoux M.-M., Danguy A. (2001). Galectin-1 is highly expressed in human gliomas with relevance for modulation of invasion of tumor astrocytes into the brain parenchyma. Glia.

[134] Roda O., Ortiz-Zapater E., Martínez-Bosch N., Gutiérrez-Gallego R., Vila-Perelló M., Ampurdanés C., Gabius H.-J., André S., Andreu D., Real F.X. (2009). Galectin-1 is a novel functional receptor for tissue plasminogen activator in pancreatic cancer. Gastroenterology.

[135] Gabius H.-J., Bodanowitz S., Schauer A. (1988). Endogenous sugar-binding proteins in human breast tissue and benign and malignant breast lesions. Cancer.

[136] Gabius H.-J., Gabius S., Zemlyanukhina T.V., Bovin N.V., Brinck U., Danguy A., Joshi S.S., Kayser K., Schottelius J., Sinowatz F. (1993). Reverse lectin histochemistry: Design and application of glycoligands for detection of cell and tissue lectins. Histol. Histopathol..

[137] Kayser K., Bovin N.V., Korchagina E.Y., Zeilinger C., Zeng F.-Y., Gabius H.-J. (1994). Correlation of expression of binding sites for synthetic blood group A-, B-, and H-trisaccharides and for sarcolectin with survival of patients with bronchial carcinom. Eur. J. Cancer.

[138] Gready J.N., Zelensky A.N., Gabius H.-J. (2009). Routes in lectin evolution: case study on the C-type lectin-like domains. The Sugar Code. Fundamentals of Glycosciences.

[139] André S., Kopitz J., Kaltner H., Villalobo A., Gabius H.-J., Gabius H.-J. (2009). Glycans as functional markers in malignancy?. The Sugar Code. Fundamentals of Glycosciences.

[140] Schwartz-Albiez R., Gabius H.-J. (2009). Inflammation and glycosciences. The Sugar Code. Fundamentals of Glycosciences.

